# Flexural Capacity of Concrete Beams with Basalt Fiber-Reinforced Polymer Bars and Stirrups

**DOI:** 10.3390/ma15228270

**Published:** 2022-11-21

**Authors:** Julita Krassowska, Carolina Piña Ramírez

**Affiliations:** 1 Department of Building Structures, Bialystok University of Technology, 15-351 Bialystok, Poland; 2 Departamento de Construcciones Arquitectonicas y su Control, Escuela Técnica Superior de Edificación, Universidad Politécnica de Madrid (UPM), Avda. Juan Herrera 6, 28040 Madrid, Spain

**Keywords:** basalt fiber-reinforced polymer, basalt fibers, basalt fiber-reinforced polymer stirrups, flexural capacity

## Abstract

The flexural properties of six 120 × 300 × 4500 mm concrete beams reinforced with bars made from basalt fiber-reinforced polymer (BFRP) basalt fibers and concrete stirrups were investigated. The beams contained different concrete compositions (with or without basalt fibers). Steel and BFRP bars were used as longitudinal and shear reinforcement. As expected, all the beams failed by the crushing of the concrete in the top compression fibers because of using BFRP bars. Beams with BFRP bars should be designed to fail by concrete crushing because it is safer than a brittle failure of the bars. The beams with composite reinforcement were characterized by the greatest number of cracks with the largest crack width. The use of basalt fibers resulted in slightly reduced cracks in beams. The most significant deflections were recorded for the beams with BFRC composite reinforcement, the smallest for FRC beams. Adding basalt fibers to the concrete resulted in slightly reduced deflection of FRC beams compared to RC beams and significantly reduced deflection compared to BFRC beams. Results showed that introducing basalt fibers to the concrete increased curvature ductility of these beams. A theoretical analysis of flexural capacity showed that the ACI standard design is more similar to experimental values (0.87). A more restrictive standard, as it turns out, is the *fib* Model Code (0.68).

## 1. Introduction

Composite bars (fiber-reinforced polymer—FRP) have been available on the international market as concrete reinforcement for 15 years. FRP is a family of materials with diverse properties that can be altered at the production stage [1]. The following can be distinguished: glass fiber (GFRP), carbon (CFRP), aramid (AFRP), and basalt (BFRP) bars [2]. Currently, due to its durability, electromagnetic neutrality, high strength, and lightness, each year over 10 million meters of this type of material is used in construction [3]. FRP bars are lightweight, corrosion-resistant, and have high tensile strength. Therefore, many researchers have used FRP bars as an alternative to the traditional steel bars in RC members subjected to monotonic and cyclic loading. Although FRP bars have several advantages, they are not widely used in construction because they are brittle and can decrease the ductility and bond strength of the RC members [4].

FRP bars are a relatively new material whose properties have not yet been thoroughly investigated. The influence of the mechanical properties of bars of this type on the load-bearing capacity and deformability of concrete construction elements containing them has also not been determined. Due to the anisotropic structure of composite materials, potentially adequate constitutive compounds need to be assumed by the anisotropic body theory. The research conducted so far has also shown that Young’s modulus of composite bars is approximately five times lower than that of steel reinforcement [5], which results in a much more significant reduction of the cross-sectional stiffness of an FRP-reinforced beam after cracking compared to a reinforced concrete beam [6]. Numerous studies have been conducted to determine the potential of FRP in RC applications. Most of them were performed on glass FRP (GFRP) [7,8], carbon FRP (CFRP) [9], and aramid FRP (AFRP) [10]. Relatively, the fewest studies presented in the literature concern the use of BFRP basalt bars [11,12,13,14]. CFRP has excellent mechanical properties and corrosion and fatigue resistance. However, the high price of carbon fiber and low elongation at break may have certain restrictions on engineering applications of concrete structures. GFRP and BFRP has lower price and higher toughness due to the abundance of raw materials and the maturity of the preparation technology. At the same time, BFRP is also a new composite developed in recent years and been proved to have good performance and can be used in concrete structures [15]. However, the low elastic modulus and linear stress–strain behavior of FRP bars often lead to large deflections, wide cracks, and brittle failure in FRP bar-reinforced concrete structures. Therefore, the serviceability limit state usually becomes the governing criterion controlling the design of FRP bar-reinforced concrete structures. Thus, FRP bars cannot reach ultimate tensile strength at failure, which limits their applications in construction projects [16].

Despite the popularization of various types of nonmetallic bars and the numerous tests performed on structural elements, this type of reinforcement is still treated as an unconventional construction material. One of the reasons is the lack of national standards and clear guidelines for the design of structures reinforced with FRP bars.

The research conducted so far has mainly focused on nonmetallic reinforcement in the form of BFRP longitudinal bars. There have been no tests of concrete beams with BFRP stirrups and with basalt fibers. In many studies, adding basalt fibers in to concrete structures improved their flexural capacity [2,17,18].

The aim of the research presented in this paper was to evaluate the behavior of flexural concrete beams reinforced with basalt bars, stirrups (BFRP), and basalt fibers compared to the behavior of beams with steel reinforcement. The values of the load-bearing capacity, deflection, and deformation of the tested elements were determined under the conditions of four-point bending. Using the available code procedures for the design of concrete elements with FRP reinforcement, theoretical calculations of the load-bearing capacity of the tested beams were carried out and verification against the experimental values performed.

## 2. Materials and Methods

Six concrete beams with a cross section of 120 × 300 mm and a length of 4500 mm were tested. They were designed with different longitudinal and shear reinforcements and with different contents of basalt fibers in the concrete mix. Table 1 presents the details of the parameters of the tested beams.

In series A, the longitudinal reinforcement was made of steel bars. In series B, basalt bars were used. In both series, the following bars were used: 2∅12 mm bars in the compression zone, steel bars 4∅14 mm in the tension zone, and 4∅14 mm basalt bars. The degree of tension reinforcement in the tested beams was $\rho_{f}$ = 2.47%, while the balanced reinforcement ratio with BFRP bars was $\rho_{fb}$ = 0.37%. The shear reinforcement was designed as stirrups with a spacing of 200 mm made from ∅6 mm steel bars in series A, while in series B, it was made from BFRP ∅6 mm bars. The shear reinforcement was designed to prevent wall failure and obtain a model of flexural failure.

### 2.1. Materials

The concrete mixture was made of CEM I 42.5R Portland cement. The selected test cement is characterized by a very high early strength. The cement content was 320 kg/m^3^. Concrete with a ratio of w/c = 0.5 was selected for the test. The used aggregate was a mixture of sand with a grain size of up to 2 mm and a coarse natural aggregate with a grain size of up to 8 mm. The fraction of up to 2 mm was 51%, the 2–4 mm fraction 38%, while the 4–16 mm fraction was 62% of the crumb pile. Detailed mixture information is given in Table 2.

**Figure 1:**
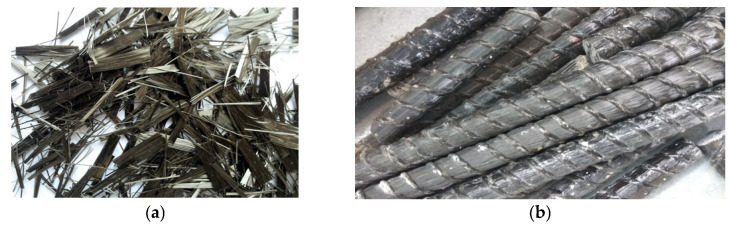
Reinforcement used in tests: (**a**) basalt fibers, (**b**) BFRP bars and stirrups.

For the purpose of the tests, basalt fibers in an amount of 8 kg/m^3^ were used as dispersed reinforcement. These are thin basalt fibers with a fiber diameter of 20 µm, a tensile strength of 750 MPa, and a Young’s modulus of 89 GPa. Part of the aggregate was replaced by a volume of the dispersed reinforcement in Figure 1.

The slump [19] and air content [20] were determined in the fresh concrete mixture. Tests of the compressive strength *f_ck_* of concrete were carried out pursuant to EN 12390-3:2011 [21] using cubic samples with a side of 100 mm, concrete flexural strength *f_ctm_* was tested on samples with dimensions of 100 × 100 × 400 mm pursuant to EN 12390-5:2011 [22], and elasticity modulus *E_cm_* was determined according to EN 12390-13:2014 [23] using cylindrical specimens with a diameter of 150 mm and a height of 300 mm. The results of concrete testing are presented in Table 3.

BFRP reinforcement was used in the form of bars with 6 mm and 14 mm diameters. For the reinforcement of the research models, ϕ6 mm and 16 mm ribbed bars were used, made of BSt500s steel with a yield point of *f_yk_* = 500 MPa. The mechanical properties of BFRP bars were tested on a bar with a diameter of 6 mm, pursuant to ACI440.3R [24], i.e., the guaranteed tensile strength was *f_u,ave_* = 1180 MPa, the guaranteed modulus of elasticity was *E_f_* = 47.6 GPa, and the guaranteed strain at break was ε*_fu_ = 2.0%. A detailed description of the research can be found in paper [25].

### 2.2. Test Setup and Testing Procedure

**Figure 2:**
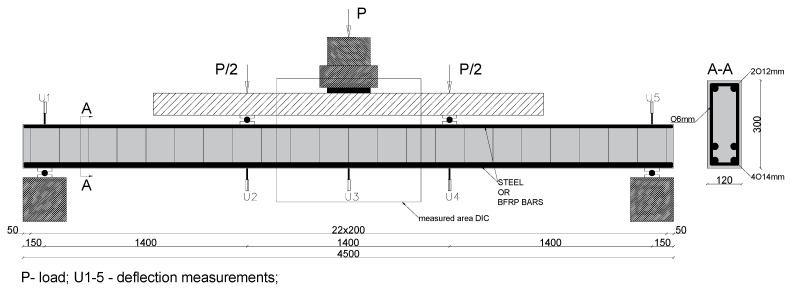
The schemes of tested beams. (P-load, U- deflection measurement place).

Simply supported beams with a span length of *l_eff_* = 4200 mm were loaded in a four-point system. The beam support and loading scheme are shown in Figure 2. The beams were hinged at one end, free to roll at the other. The span length of the pure-bending region between the two load points was 1400 mm. The two-point load setup consisted of a wide flange beam spread on two steel plates covering its entire width. To enable their rotation, the steel plates had steel rollers. The test of the beams was load-controlled at a rate of 5 kN per minute. The load was generated by a hydraulic jack located at the center of the beam and applied to the broad flange. A load cell attached to the hydraulic jack measured the applied load. The mid-span and support deflections (U) were measured using a linear variable differential transformer. Mid-span deformations were measured using DIC (digital image correlation). The measurement set consists of two cooperating cameras and a control unit, enabling the recording of up to 5 million readings at a frequency of 15 Hz. The measurement procedure consists in the initial calibration of cameras for a given working area, applying a pattern, and ultimately recording the displacement of measurement points during the so-called facets.

## 3. Results

This section provides a summary of the overall flexural behavior of BFRP PC beams in terms of their failure mode, crack pattern and distribution, load-deflection behavior, strain on the surface of concrete in the compression and tension zones, DIC maps, and the ultimate flexural capacity.

### 3.1. Failure Modes

**Figure 3:**
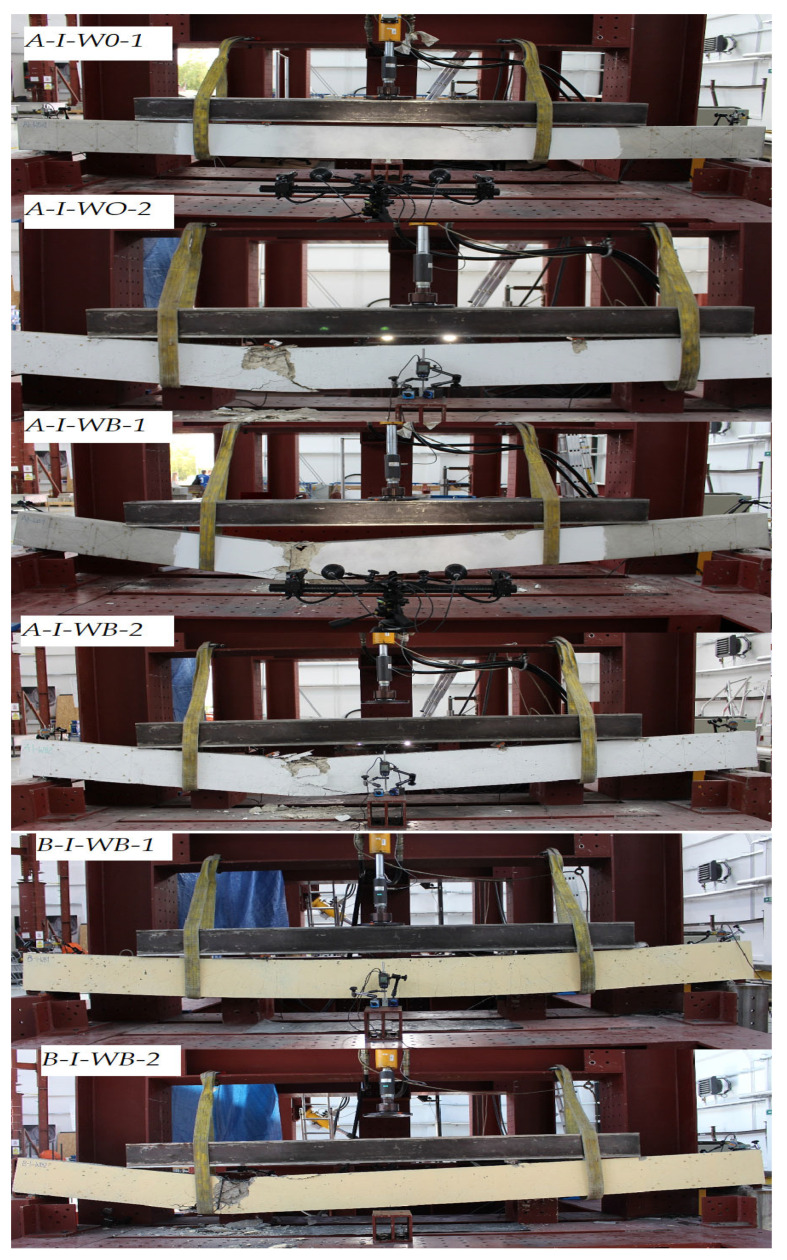
Failure mode of RC and BFRC RC beams.

In line with the assumptions, the beams failed at bending (Figure 3). All the beams showed a similar pattern of failure, i.e., the crushing of concrete in the compression zone. One of the beams in series B-I-WB1 achieved the highest deflection, which resulted from its leaning against the belts and thus changing the static pattern of the beam (the test was discontinued at that point). The beams with composite reinforcement were characterized by the greatest number of cracks with the largest crack width. The use of basalt fibers resulted in slightly reduced scratching of the beam’s cross section.

**Figure 4:**
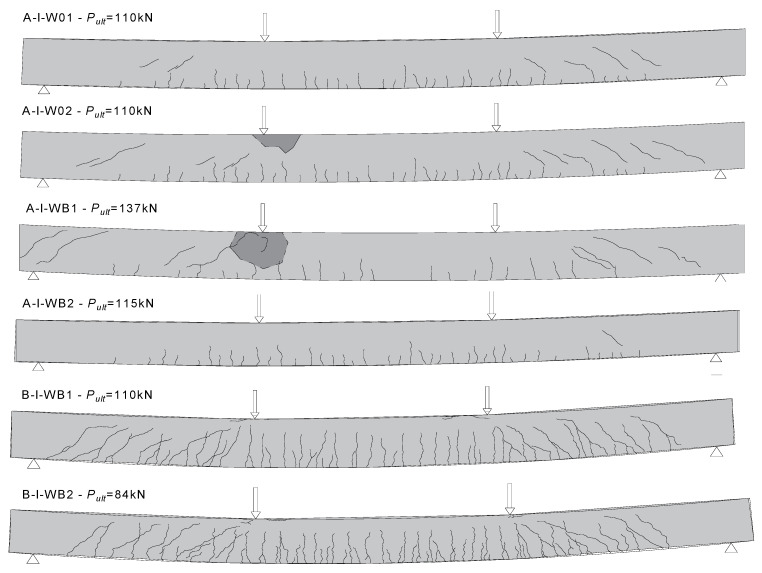
Crack patterns of the tested beams at failure.

The cracking patterns for the eight RC beams are shown in Figure 4. In the case of BFRC RC beams, the first perpendicular crack developed at 0.1 *P_ult_*. The first vertical flexural crack in BFRC RC beams was initiated in the middle of the constant moment region. The diagonal cracks formed when a load of approximately 0.5 *P_ult_* was applied. The first perpendicular cracks in RC and FRC reference beams developed when a load of 0.2 *P_ult_* was applied. Diagonal cracks formed much later in RC (0.45 *P_ult_*—A-I-W01, 0.45 *P_ult_*—A-I-W02) and FRC (0.60 *P_ult_*—A-I-W01, 0.52 *P_ult_*—A-I-W02) beams. This means that beams with steel reinforcement and basalt fibers had the highest scratch resistance. BFRC RC beams showed the lowest scratch resistance.

Beyond the first cracking load, additional flexural cracks developed along the beam length within the constant moment region. With increased loading, flexural cracks propagated toward the top fiber and new cracks started to develop in the shear region. The cracks that developed outside the pure-bending region were inclined. The beam continued to sustain the load until it reached the maximum flexural capacity, at which point the concrete in the constant moment region at the compression zone crushed. Beams with the higher reinforcement ratio showed a lower number of cracks and smaller crack depths within the constant moment region, but a deeper crushed section in the compression zone at failure, as shown in Figure 4. After failure, all the beams recovered most of the deflection when unloaded, as the strain in the bars did not reach the ultimate rupture strain. This behavior is expected in FRP RC beams failing by the crushing of concrete. A small degree of permanent deflection remained in the beams due to the damage caused by the crushing of concrete and the possible bond slippage between the BFRP bars and concrete.

Table 4 shows a summary of the failure forces for the individual beams *P_ult_* and the ultimate flexural moments *M_ult_*, along with the average values of the failure force $\bar{P_{ult}}$, the ultimate bending moment $\bar{M_{ult}}$, and the change in the value of forces $\Delta P_{ult}$, with the destructive force specified for the reference A-I-W0 beams. The manner of beam failure and the maximum deflections are also shown.

The introduction of dispersed reinforcement in the form of basalt fibers increased the destructive force (A-I-WB). The value of the breaking force determined for series B-I-WB beams was slightly lower compared to the destructive force in series A-I-W0. The use of basalt fibers reduced the number of cracks on the beams. However, a significantly greater number of cracks were observed for Lek with BFRP reinforcement, which exhibited better formability. Mainly perpendicular cracks developed, caused by flexure. Under destructive loads, steel reinforcement was subject to local adhesion loss, while composite reinforcement developed brittle fractures.

### 3.2. Moment-Deflection Behavior

**Figure 5:**
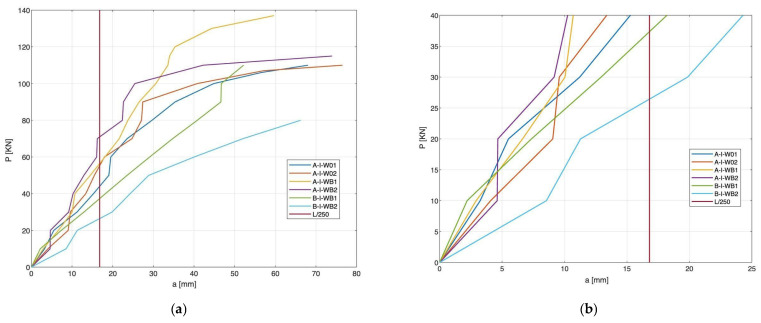
Load-deflection curves for the tested beams. (**a**) Relationship until ultimate deflection; (**b**) Relationship between the development of the first crack and deflection limits.

Figure 5 shows diagrams of average deflection mid-span *a* [mm], depending on the value of force P [kN]. The charts show temporary deflections in relation to the positioning of supports.

Beams with BFRP reinforcement showed the highest deflection values and the lowest FRC beams. Adding basalt fibers to concrete resulted in a slight reduction in the deflection of FRC beams compared to RC beams and a significant reduction compared to BFRC beams. In the initial phase of beam operation with a load of 10 kN, FRC beams showed a deflection value similar to that of RC, while in the case of BFRC beams, deflection was 43% greater than for RC beams. In the case of BFRC beams, the first perpendicular cracks appeared at a deflection value of approximately 10 mm, in the case of RC beams 34 mm, and in the case of FRC beams 40 mm. As the load increased, BFRC beams invariably showed a more significant deflection. A comparison of the curves presented in Figure 4 shows that an appropriate proportion of dispersed reinforcement in the concrete may reduce beam deflection. In the case of concrete beams with basalt fibers, a nonlinear relationship was observed, with a gradual decrease in the slope of the plot until failure. The use of fibers reduced the deflection of the beams due to the tension-stiffening effect.

According to the EN2 standard, the maximum permissible deflection value in the serviceability limit state is L/250. In the series where the BFRP reinforcement was used, the deflection was exceeded at a force of 30 kN, for reinforced concrete beams 48 kN, and in the case of FRC beams 70 kN. The concrete with basalt fibers used in the tested beams increased the stiffness, while BFRC beams showed the lowest stiffness. BFRC beams achieved the greatest deflections, while the reduced stiffness was connected with the lower modulus of elasticity of BFRP bars. The use of basalt fibers in the beams reduces deflection. The use of a combination of basalt composite reinforcement and concrete with basalt fibers reduced the effect of decreased stiffness of BFRC beams.

### 3.3. Strain in Concrete in Compression and Tension Zones

**Figure 6:**
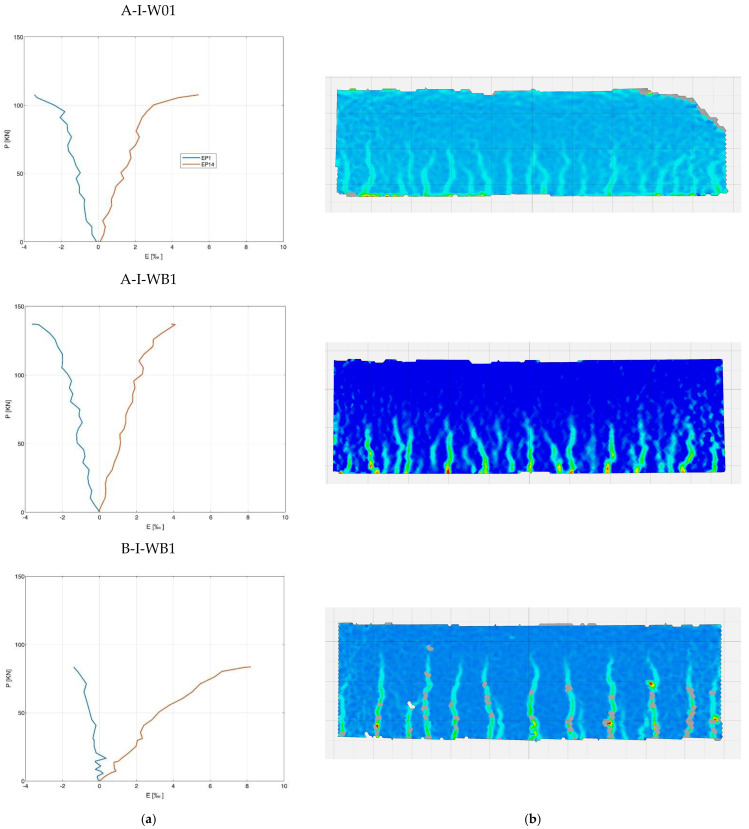
Strains in concrete in series A-I-W01, A-I-WB1, and B-I-WB1. (**a**) The position of longitudinal reinforcement in tension and compression zones; (**b**) Strains in the DIC area.

Figure 6 shows the maps of the main deformations, obtained using DIC (digital image correlation system) and diagrams of deformations on the concrete surface at the level of the longitudinal compression and tension reinforcement. The analysis of the deformation maps of the individual series corresponds to the recorded scratching, as shown in Figure 3. Perpendicular cracks, typical for flexural elements, can be seen at the places of the maximum moment values, regardless of the test series. In the reference series and series with basalt fibers, the height of the cracks did not exceed half of the height of the beam; hence, it can be assumed that there exists a neutral axis approximately mid-height. In the case of the beams with BFRP reinforcement, cracks reach over the midpoint of the beam’s height, which means that there is a shift in the section’s neutral axis. An analysis of the values of deformation on the concrete surface at the levels of compression and longitudinal tension reinforcements confirms the significant increase in tensile strains at the level of BFRP reinforcement compared to steel reinforcement, and a decrease in the value of strains in the compression zone.

Table 5 presents a quantitative summary of the values of deformation in the individual series. The deformation in the compression zone in series A-I-WB just before failure and at a breaking force at half of its maximum value increases up to 10% compared to the reference series. Composite beams in the compression zone show a reduction in the deformation values just before failure and at a breaking force at half of its maximum value, by 60% and 70% compared to the reference series, and by 70% in 0.5 *P_ult_* and 60% in *P_ult_* compared to the series with steel reinforcement and basalt fibers, i.e., A-I-WB. On the other hand, the composite beam reaches the highest deformation value in the tensile zone of the beam under breaking load, equal to 8‰. This value is half that of the steel reinforcement or the basalt fibers series.

## 4. Comparison of the Calculated and Experimental Values of Flexural Capacity

Guidelines ACI 440: 1R-06 (2006) and *fib* Model Code 2010 were adopted as the basis for the theoretical analysis of the test results.

The ACI method [26] is one of the most frequently used calculation procedures for structures with nonmetallic reinforcement. It employs the conventional methods of deformation compliance, where excellent adhesion between the BFRP bars and the concrete is assumed. The methods of equilibrium are used for analyzed concrete sections with the BFRP bars. The analysis of the ultimate moment of load-bearing capacity is based on the consideration of the linearly elastic behavior of the BFRP bars.

**Figure 7:**
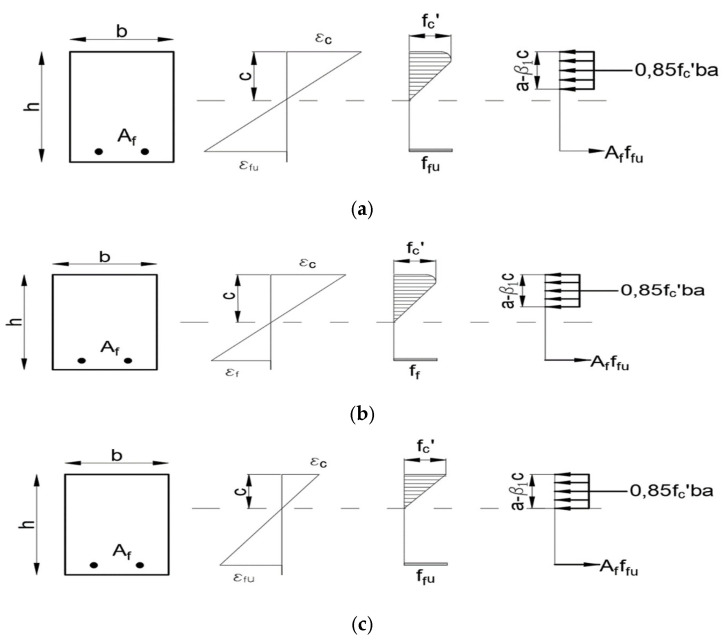
Three operating states of the structure with BFRP reinforcement: (**a**) cross section with the optimal degree of reinforcement, (**b**) rearmed cross section—failure by the crushing of concrete, (**c**) underreinforced cross section—failure by bars breaking.

Based on Figure 7, three basic operating states of a structure with BFRP reinforcement can be seen.

The condition determines the ultimate limit state for flexure:

(1) $$\phi M_{n}\geq M_{u},$$

where *M_n_* is the flexural capacity, *M_u_* is the maximum moment due to external loads, while ϕ is the reduction factor. The application of factor ϕ is due to the lack of plastic deformation of BFRP reinforcement.

**Figure 8:**
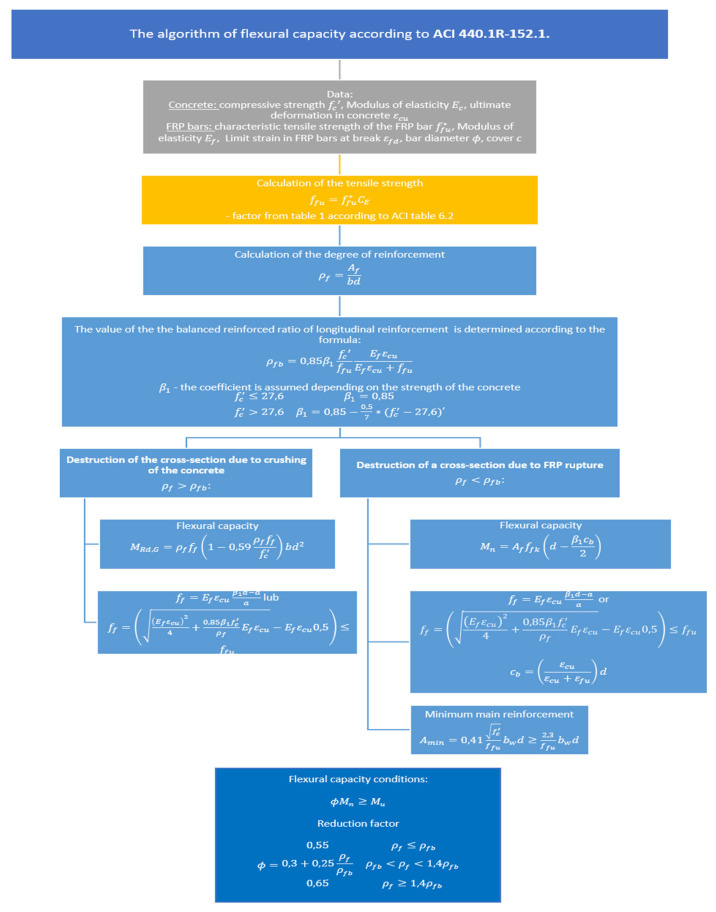
The algorithm of flexural capacity pursuant to the ACI guidelines.

Figure 8 shows the algorithm of cross-section dimensioning according to ACI.

As mentioned, under flexural moment, beams with steel reinforcement simultaneously plasticize the reinforcement in the tension zone and crush the concrete in the compression zone. This is the optimal failure model, and the cross section is thus called “optimal.”

In the case of a linear σ–ε relationship of FRP bars, a situation whereby the cross section is destroyed by the concrete being crushed under compression is aimed for. Breakage of FRP bars occurs when the degree of reinforcement is less than optimal, i.e., the cross section is underreinforced. This occurs when the cross section is reset, i.e., the degree of reinforcement of the cross section is greater than optimal. The model of this type of failure is believed to be safer than the fracture of FRP bars.

The application of factor *ϕ* is due to the lack of plastic deformation of the BFRP reinforcement. The assumed values of the reduction factor of the load-bearing capacity range from 0.65 to 0.55, to account for the uncertainty related to the varying degrees of reinforcement.

**Figure 9:**
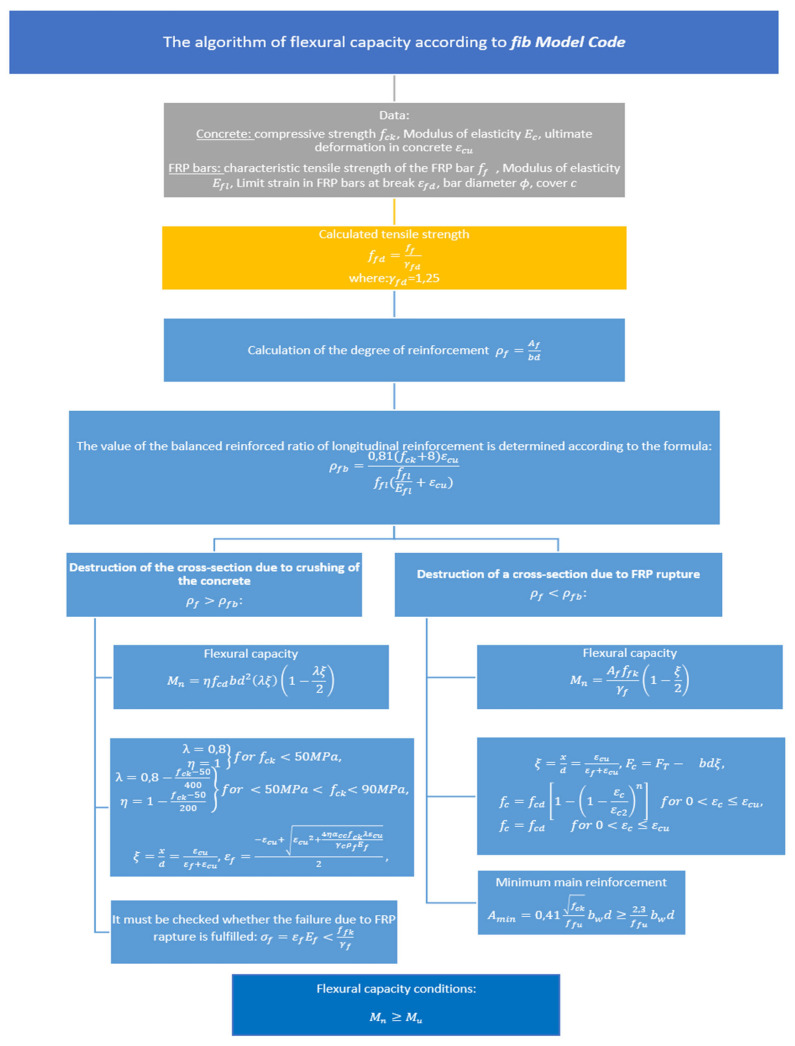
The algorithm of flexural capacity pursuant to the *fib* Model Code guidelines.

The basis for the *fib* Model Code [19] is the paper by Triantafillou et al. [27]. Figure 9 shows the algorithm for the dimensioning of FRP cross sections according to this standard.

The parameters of FRP composite bars are tensile strength $f_{f}$ and percentage of total elongation $f_{k}$ at maximum force $\varepsilon_{fuk}$.

Given the generally limited compressive modulus and the risk of microbuckling or displacement of the fiber within the restraint of the matrix material, nonmetallic reinforcement is generally not expected to withstand high compressive stresses. Nonmetallic reinforcement is characterized by excellent chemical resistance and insensitivity to a wide range of aggressive media.

**Figure 10:**
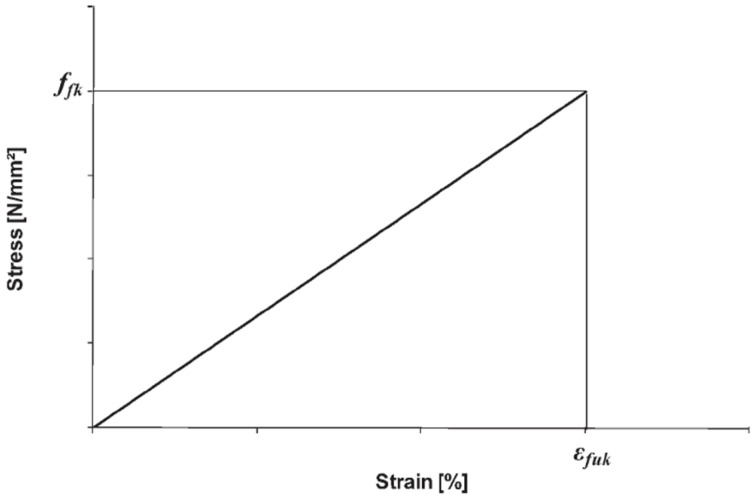
Idealized stress–strain diagram for BFRP bars.

For design purposes, an idealized stress–strain diagram (Figure 10) is to be used.

The purely linear elastic nature of the above relationship significantly influences the change of the design approach used in determining the load-bearing capacity of concrete elements reinforced with steel bars. Similarly to the case of the ACI code, two models of structural failure are assumed, i.e., concrete crushing $\rho_{f}>\rho_{fb}$ and failure by breaking the bars $\rho_{f}<\rho_{fb}$.

**Figure 11:**
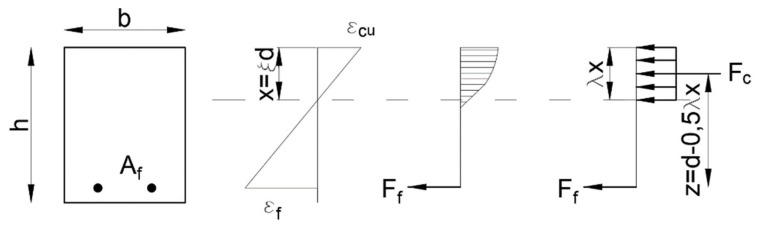
Equilibrium conditions of the FRP cross section.

The method for the determination of flexural capacity is based on the assumptions of pre-code EC2 (Figure 11). The compressive strength of FRP bars is ignored.

Table 6 shows the results of the analysis of the flexural capacity of the tested beams, where *M_ult_* is the bending resistance determined from scientific studies. The flexural capacity was determined according to the ACI *M_ACI_* and *fib* Model Code guidelines for *M_fib_.*

Comparison of the theoretical and experimental values of the bending resistance of the tested beams shows that the ACI standard is the closest to the experimental values (0.87). The more restrictive standard, it turns out, is the *fib* Model Code [19] (0.68).

Flexural capacity depends on the compressive strength of the concrete, the amount of FRP reinforcement, and its strength. In the case of the *fib* Model Code [27], it also depends on the extent of the compression zone in the concrete. The differences in the magnitude of the flexural capacity depend on the following:

- the methods for the determination of the tensile strength of FRP reinforcement, often depending on environmental factors;
- the reduction coefficients of the load-bearing capacity, depending on the compressive strength of the concrete;
- the methods for calculating the extent of the compression zone;
- the different values of the ultimate deformation of concrete.

The calculations also show differences in the values of the balanced reinforcement ratio determined pursuant to the norms mentioned above. Depending on the value of the balanced reinforcement ratio, it can be determined whether the cross section is destroyed by the crushing of concrete or by the breaking of FRP bars. Theoretically, regardless of the standard, all beams unambiguously meet the conditions of failure by the crushing of concrete, which corresponds to the model of failure of the tested beams. The differences in the values are mainly due to the method of determination of the balanced reinforcement ratio and the coefficients used in the formula, which depend on the compressive strength of the concrete. The value of the balanced FRP reinforcement ratio increases with increasing compressive strength and decreases with increasing tensile strength of FRP bars. A higher value of the degree of reinforcement ratio is obtained in the *fib* Model Code, while the lowest value is observed in the ACI. In theory, this means that above this value, failure occurs by the breaking of the bars.

## 5. Conclusions

The ultimate flexural behavior of six full-scale beams reinforced with different bars, steel, or BFRP and basalt fibers was investigated. Based on the test results and the analytical investigation, the following conclusion can be drawn.

1. As expected, the six beams with BFRP bars and stirrups failed by the crushing of concrete at mid-span in the compression fibers.
2. The presence of composite reinforced bars increases the deformation value due to tensile stresses. Using the FRP reinforcement resulted in improving the flexural capacity of beams, regardless of the concrete type.
3. Due to the relatively low value of Young’s modulus of BFRP reinforcement, the stiffness of the beam decreases significantly after scratching. After the drawing moment is exceeded, perpendicular cracks of considerable width are formed in the beam’s central section at the tension reinforcement level. Due to the corrosion resistance of BFRP bars, the crack width is not as important as in the case of reinforced concrete structures.
4. BFRP basalt bars also influenced the nature of beam failure, which did not occur suddenly, but was rather associated with the forming of many cracks and significant deflection of the element.
5. The methods for designing flexural capacity are based on the equations of the equilibrium of forces and moments in the cross section, as is the case with steel members.
6. During the design, the differences resulting from the different physical and mechanical properties of the BFRP reinforcement compared to the steel should be considered. For this reason, rectangular stress distribution is assumed in the compression zone.
7. The analysis showed differences of approximately 20% in the flexural capacity of the beams. The differences are mainly due to the use of different reduction factors. The difference in the results would have been much more significant when determining the flexural capacity using material factors that are ignored when compared with the test results.

## Figures and Tables

**Table 1:** Beam designation and reinforcement parameters of BFRP RC beams.

Beam No.		Bar Diameter, mm	No. of Bars	Materials of Bars	Basalt Fibers, kg/m^3^
RC	A-I-WO1	14	4	steel	0
	A-I-WO2	14	4	steel	0
FRC	A-I-WB1	14	4	steel	8
	A-I-WB2	14	4	steel	8
BFRC	B-I-WB1	14	4	basalt	8
	B-I-WB2	14	4	basalt	8

**Table 2:** Mixture proportions for the concrete.

Mixture Proportions	Quantity
Cement 42.5R, kg/m^3^	320
Water, kg/m^3^	160
Sand 0.125–4 mm, kg/m^3^	732
Aggregate, kg/m^3^	1203

**Table 3:** Measured concrete parameters.

Fiber Content [kg/m^3^]	Slump	Air Content	*f_ck_*	*σ*	*ν*	*f_ctm_*	*σ*	*ν*	*E_cm_*
	mm	%	MPa	MPa	%	MPa	MPa	%	GPa
0	19	3.1	43.78	0.6	0.96	5.55	0.85	15.28	40.64
8	2	3.8	44.52	2.75	6.17	6.11	0.68	12.14	42.02

**Table 4:** Results of tests of beams.

Beam No.		Max. Load *P_ult_*	$\bar{P_{ult}}$	Moment *M_ult_*	$\bar{M_{ult}}$	Failure Mode	Ultimate Deflection ∆*a_lim_*
		kN	kN	kNm	kNm	-	mm
RC	A-I-W01	110	110	77	77	F	59.67
	A-I-W02	110		77		F + CC	73.97
FRC	A-I-WB1	137	126	95.9	88.2	F + CC	68.04
	A-I-WB2	115		80.5		F + CC	76.52
BFRP	B-I-WB1	110	97	58.8	67.9	F	52.21
	B-I-WB2	84		77		F + BR	52.06

F—flexure; CC—concrete crushing; BR—bar rupture.

**Table 5:** Strain in concrete.

Beam No.		Load	Strain in Concrete on the Position of Longitudinal Reinforced	
			In compression *ε*	In tension *ε*
		[-]	[‰]	[‰]
RC	A-I-W0	*0.5 P_ult_*	−1.050	1.513
		*P_ult_*	−3.443	5.420
FRC	A-I-WB	*0.5 P_ult_*	−1.153	1.339
		*P_ult_*	−3.632	4.076
BFRP	B-I-WB	*0.5 P_ult_*	−0.332	2.356
		*P_ult_*	−1.533	8.838

**Table 6:** Experimental *M_ult_* and the designed load-bearing capacity of beams.

Load-Bearing Capacity		RC	BFRC
*M_ult_*	kNm	82.50	76.50
*M_ACI_*	kNm	107.98	66.82
*M_ult_/M_ACI_*	-	1.31	0.87
*M_fib_*	kNm	76.97	52.01
*M_ult_/M_fib_*	-	0.93	0.68

## Data Availability

The data presented in this study are available on request from the corresponding author.
